# Covid-19 em Receptores de Transplante de Coração em São Paulo: Uma Série de Casos

**DOI:** 10.36660/abc.20200722

**Published:** 2021-02-02

**Authors:** Rafaela Vale de Miranda Soriano, João Manoel Rossi, Marco A. Finger, Carolina Casadei Santos

**Affiliations:** 1 Instituto Dante Pazzanese de Cardiologia São PauloSP Brasil Instituto Dante Pazzanese de Cardiologia, São Paulo, SP - Brasil

**Keywords:** Doenças Cardiovasculares/cirurgia, Transplante do Coração/complicações, Coronavírus, Betacoronavírus, Covid-19, Síndrome Respiratória Aguda, SARS-CoV2, Transplantados, Inflamação

## Introdução

A doença causada pelo novo coronavírus (Covid-19), o SARS-CoV-2 (*severe acute respiratory syndrome coronavirus 2*), foi declarada uma pandemia pela Organização Mundial da Saúde (OMS) em 11 de março de 2020.^1^ Por analogia a outras infecções respiratórias, principalmente com base na pandemia de 2009 do vírus influenza H1N1,^2^^,^^3^ esperava-se um aumento de casos de pneumonia e progressão para choque séptico com síndrome do desconforto respiratório agudo entre os receptores de transplante de órgão sólido com Covid-19 em comparação com a população não transplantada.^4^ Porém, a imunossupressão no transplante poderia teoricamente revogar a síndrome hiperinflamatória secundária à tempestade de citocinas, responsável pela maioria das mortes pela Covid-19.^5^^,^^6^ Dados sobre a imunossupressão que potencialmente poderiam levar a apresentações clínicas atípicas ou aumentar o risco de eventos adversos na presença de Covid-19 são conflitantes.^7^^,^^8^

Relatamos nossa experiência com transplantados de coração (TxC) diagnosticados com Covid-19 em uma instituição com programa de TxC desde 1992 em São Paulo, Brasil.

## Material e Métodos

### População e Cenário

Receptores de TxC adultos atendidos no Instituto Dante Pazzanese de Cardiologia entre março e junho de 2020, com sinais e sintomas sugestivos de infecção por SARS-CoV-2 e que testaram positivo para reação em cadeia da polimerase com transcriptase reversa (RT-PCR), ou com achados radiológicos compatíveis com Covid-19.

Os dados foram coletados em revisão de prontuários. Foram incluídos história clínica, resultados laboratoriais, marcadores inflamatórios e radiológicos, e terapias administradas. Descrevemos óbito por Covid-19, admissão na Unidade de Terapia Intensiva (UTI), necessidade de ventilação mecânica e disfunção renal.

### Métodos Estatísticos

Os resultados foram relatados de forma descritiva.

## Resultados

Cinco pacientes de TxC foram hospitalizados por Covid-19. Nenhum deles foi diagnosticado por triagem assintomática.

A idade variou de 35 a 79 anos. As comorbidades foram diabetes mellitus (DM) (100%), hipertensão arterial (HAS) (80%), doença renal crônica (40%) e obesidade (20%). O tempo de TxC foi de três meses a 264 meses. Foram administrados inibidores da calcineurina em quatro pacientes (80%), inibidor da mTOR em 40%, e prednisona e micofenolato em 100% dos pacientes. Os sintomas foram febre documentada (80%), tosse na admissão (100%), dispneia (60%) e sintomas gastrointestinais (20%) (Tabela 1).

**Tabela 1:** Dados epidemiológicos e sintomas associados à infecção por SARS-CoV-2 em TxC

Pacientes	Idade (anos)	Sexo	Tempo de transplante (meses)	Comorbidades	Imunossupressão	Sintomas
1	79	Masc.	264	HAS, DM, DRC	IC, AM, CE	Febre, tosse, dispneia
2	67	Masc.	264	HAS, DM, DRC	imTOR, AM, CE	Tosse, dispneia
3	52	Fem.	192	HAS, DM, Obesidade	IC, AM	Febre, tosse, dispneia
4	50	Masc.	84	HAS DM	IC, imTOR, AM, CE	Febre, tosse, SGI
5	35	Fem.	3	DM	IC, AM, CE	Febre, tosse

Masc: masculino; Fem: feminino; HAS: hipertensão arterial sistêmica; DM: diabetes mellitus; DRC: doença renal crônica; IC: inibidor da calcineurina; imTOR: inibidor da mTOR; AM: antimetabólico; CE: corticoesteroide; SGI: sintomas gastrointestinais.

Conforme Tabela 2, ocorreu linfopenia (<1,500 mm³) em todos os pacientes e trombocitopenia (<150,000 mm³) em 60% deles. A troponina estava elevada em um dos casos de óbito, enquanto no outro não foi coletada. Também foi registrada alteração no lactato em um paciente que evoluiu a óbito. Marcadores inflamatórios aumentados foram comuns, sendo mais altos naqueles que necessitaram de cuidados intensivos. Foi realizada tomografia de tórax em todos os pacientes, que apresentaram infiltrados pulmonares bilaterais em vidro fosco. Insuficiência renal esteve presente em 80%.

**Tabela 2:** Dados laboratoriais associados à infecção por SARS-CoV-2 em TxC

Paciente	Leucócitos Totais (mm³)	Linfócitos totais (mm³)	Plaquetas <150000/mm³	IRA	Trop I (ng/ml)	Dim-D (mg/L)	Lactato >2mmol	PCR	DHL	BNP
1	6.100	670	Sim	Sim	NC	1836	Não	20	397	NC
2	12.570	570	Sim	Sim	0.41	1.397	Sim	40	348	7.441
3	3.760	960	Não	Não	0.02	287	Não	7.1	339	1.230
4	7.760	1.300	Sim	Sim	0.01	nc	Não	0.5	NC	NC
5	8.350	420	Não	Sim	0.03	675	Não	1.1	NC	2.800

IRA: insuficiência renal aguda; Trop: troponina; Dim: dímero; PCR: proteína C-reativa; DHL: Desidrogenase láctica; BNP: Peptídeo natriurético; NC: não coletado.

Conforme descrito na Tabela 3, dois pacientes não receberam terapias empíricas para a Covid-19. Vasopressores e ventilação mecânica foram necessários em 20% dos pacientes. Nenhum paciente recebeu oxigenação por membrana extracorpórea. Nenhum paciente ficou na posição pronada e o tempo de internação na UTI foi quatro dias para ambos que necessitaram desse cuidado.

**Tabela 3:** Diagnóstico, terapêutica e desfechos associados à infecção por SARS-CoV-2 em TxC

Pacientes	Diagnóstico	TC de tórax	Óbito	Tempo de internação (dias)	UTI	DVA	VM	Terapêutica
1	RT-PCR	<50%	Sim	4	Sim	Não	Não	Azitromicina,
2	RT-PCR	>50%	Sim	4	Sim	Sim	Sim	HCQ, Azitromicina, CE
3	RT-PCR	<50%	Não	11	Não	Não	Não	NR
4	TC de tórax	<50%	Não	5	Não	Não	Não	Azitromicina
5	RT-PCR	<50%	Não	21	Não	Não	Não	NR

RT-PCR: reação em cadeia da polimerase para SARS-CoV 2; UTI: Unidade de Terapia Intensiva; DVA: droga vasoativa; VM: ventilação mecânica; HCQ: hidroxicloroquina; CE: corticosteroide; NR: não realizado; TC: tomografia computadorizada.

Os imunossupressores foram interrompidos em um paciente devido à gravidade do caso. Dois pacientes evoluíram a óbito (40%), e o restante recebeu alta hospitalar. O tempo de internação variou entre 4 e 21 dias.

## Discussão

Esta é a primeira descrição de uma série de casos de uma coorte de pacientes transplantados do coração que foram hospitalizados por Covid-19 no Brasil.

Esses pacientes parecem apresentar a Covid19 de forma semelhante a pacientes não transplantados, compartilhando os sintomas mais comuns de febre, tosse e falta de ar. Contrastando com o estudo de Scott,^9^ sintomas gastrointestinais foram observados em apenas um paciente (20%).

Todos os TxC que foram acometidos pela Covid-19 necessitaram de internação, sendo que o DM estava presente em 100% deles e HAS em 80%. Na nossa amostra, 40% dos pacientes necessitaram de cuidados intensivos e tinham o Dímero-D ≥1000 mg/L e PCR ≥20. BNP mostrou-se elevado e DHL não apresentou elevação importante quando medidos. O paciente com troponina elevada apresentou instabilidade hemodinâmica, necessidade de vasopressores e evolução para óbito, o que corrobora a literatura de que lesão miocárdica está associada a piores prognósticos.^10^ Os pacientes que foram para a UTI eram idosos, com tempo de transplante cardíaco maior, e vieram a falecer. Esses dados sugerem uma taxa de mortalidade de transplantados acima da taxa da população geral infectada pela Covid-19.^11^

As taxas de linfopenia e trombocitopenia foram mais altas neste estudo quando comparadas com relatos anteriores nas populações não transplantada e transplantada.^12^^,^^13^ Este achado poderia ser explicado por uma menor contagem basal de linfócitos e plaquetas devido ao uso de imunossupressores ou representar uma provável interferência adicional da infecção por SARS-CoV-2.

Nosso estudo tem várias limitações comuns aos estudos de TxC: o fato de ter sido realizado em um único centro e o tamanho da nossa amostra, que poderia ser considerado pequeno. Não foi possível tirar conclusões sobre tratamentos específicos para a Covid-19 ou o manejo da imunossupressão nesse cenário. Isso limita nossa compreensão sobre o espectro de sintomas e a gravidade da doença entre TxC com Covid-19.

## Conclusão

Nesta série de casos de pacientes transplantados do coração com Covid-19 atendidos em nossa instituição, a possibilidade teórica de que a imunossupressão poderia revogar a síndrome hiperinflamatória não se mostrou verdadeira. Do ponto de vista observacional, a grande quantidade de fatores de risco e a taxa de mortalidade elevada sugerem que esses receptores poderiam ser particularmente vulneráveis à Covid-19. Estudos maiores e multicêntricos são necessários para confirmar nossos achados.
