# Supplementary material for: Covid-19 em Receptores de Transplante de Coração em São Paulo: Uma Série de Casos
Source: Arq Bras Cardiol. 2021 Feb 2;116(2 Suppl 1):1–3. [Article in Portuguese] doi: 10.36660/abc.20200722 (PMC8118634; doi:10.36660/abc.20200722)
Supplement: Translation

# Translation: Covid-19 in Heart Transplant Recipients in São Paulo: A Case Series

**Authors:** Rafaela Vale de Miranda Soriano, João Manoel Rossi, Marco A. Finger, Carolina Casadei Santos

**Affiliations:** 1 São PauloSP Brazil Instituto Dante Pazzanese de Cardiologia, São Paulo, SP - Brazil

**Keywords:** Cardiovascular Diseases/surgery, Heart Transplantation: Coronavirus, Betacoronavirus, Covid-19, Severe Acute Respiratory Syndrome, SARS-CoV2, Transplant Recipients, Inflammation

## Introduction

The disease caused by the new coronavirus (Covid-19), SARS-CoV-2 (severe acute respiratory syndrome coronavirus 2), was declared a pandemic by the World Health Organization (WHO) on March 11, 2020.^1^ By analogy to others respiratory infections, mainly based on the 2009 pandemic of the influenza virus H1N1,^2^^,^^3^ an increase in cases of pneumonia and progression to septic shock with acute respiratory distress syndrome was expected among recipients of solid-organ transplantation with Covid-19 in comparison with the non-transplanted population.^4^ However, immunosuppression in transplantation could theoretically revoke the hyperinflammatory syndrome secondary to the cytokine storm, responsible for the majority of deaths by Covid-19.^5^^,^^6^ Data on immunosuppression potentially leading to atypical clinical presentations or increasing the risk of adverse events in the presence of Covid-19 are conflicting.^7^^,^^8^

We report our experience with heart transplant (HT) recipients diagnosed with Covid-19 at an institution with an HT program since 1992 in São Paulo, Brazil.

## Material and Methods

### Population and Scenario

Adult HT recipients seen at the Dante Pazzanese cardiology institute between March and June 2020, with signs and symptoms suggestive of SARS-CoV-2 infection and who tested positive for polymerase chain reaction with reverse transcriptase (RT-PCR), or with radiological findings compatible with Covid-19.

Data were collected from medical records. Clinical history, laboratory results, inflammatory and radiological markers, and therapies administered were included. We describe death by Covid-19, admission to the Intensive Care Unit (ICU), need for mechanical ventilation, and renal dysfunction.

### Statistical Methods

Results are reported in a descriptive manner.

## Results

Five HT patients were hospitalized due to Covid-19. None of them were diagnosed by asymptomatic screening.

The age ranged from 35 to 79 years. Comorbidities were diabetes mellitus (DM) (100%), systemic arterial hypertension (SAH) (80%), chronic kidney disease (40%) and obesity (20%). HT time ranged from 3 to 264 months. Calcineurin inhibitors were administered to four patients (80%), mTOR inhibitor to 40% of them, and prednisone and mycophenolate to 100%. The symptoms were documented fever (80%), cough on admission (100%), dyspnea (60%), and gastrointestinal symptoms (20%) (Table 1).

**Table 1:** Epidemiological data and symptoms associated with SARS-CoV-2 infection in HT recipients

Patients	Age (years)	Sex	Time of transplantation (meses)	Comorbidities	Immunosuppression	Symptoms
1	79	Male	264	SAH, DM, CKD	CI, AM, CE	Fever, cough, dyspnea
2	67	Male	264	SAH, DM, CKD	imTOR, AM, CE	Cough, dyspnea
3	52	Female	192	SAH, DM, Obesity	CI, AM	Fever, cough, dyspnea
4	50	Male	84	SAH DM	CI, imTOR, AM, CE	Fever, cough, GIS
5	35	Female	3	DM	CI, AM, CE	Fever, cough

SAH: systemic arterial hypertension; DM: diabetes mellitus; CKD: chronic kidney disease; CI: calcineurin inhibitor; imTOR: mTOR inhibitor; AM: antimetabolic; CE: corticosteroid; GIS: gastrointestinal symptoms.

According to Table 2, lymphopenia (<1,500 mm³) occurred in all patients and thrombocytopenia (<150,000 mm³) in 60% of them. Troponin was elevated in one case of death, while in the other it was not assessed. There was also a change in lactate in a patient who died. Increased inflammatory markers were common, being higher in those who required intensive care. Chest computed tomography (CT) scan was performed in all patients, who had bilateral pulmonary infiltrates with a ground-glass appearance. Renal failure was present in 80% of the sample.

**Table 2:** Laboratory data associated with infection by SARS-CoV-2 in HT recipients

Patient	Total leucocytes (mm³)	Total lymphocytes (mm³)	Platelets <150000/mm³	ARF	Trop I (ng/ml)	Dim-D (mg/L)	Lactate >2mmol	CRP	LDH	BNP
1	6,100	670	Yes	Yes	NC	1,836	No	20	397	NC
2	12,570	570	Yes	Yes	0.41	1,397	Yes	40	348	7,441
3	3,760	960	No	No	0.02	287	No	7.1	339	1,230
4	7,760	1,300	Yes	Yes	0.01	NC	No	0.5	NC	NC
5	8,350	420	No	Yes	0.03	675	No	1.1	NC	2,800

ARF: acute renal failure; Trop: troponin; Dim: dimer; CRP: C-reactive protein; LDH: lactic dehydrogenase; BNP: B-type natriuretic peptide; NC: not collected.

As described in Table 3, two patients did not receive empirical therapies for Covid-19. Vasopressors and mechanical ventilation were required in 20% of patients. None of them received extracorporeal membrane oxygenation. No patient was put in prone position and the length of stays at the ICU was four days for both patients who needed this care.

**Table 3:** Diagnosis, therapy and outcomes associated with infection by SARS-CoV-2 in HT recipients

Patients	Diagnosis	Chest CT	Death	Length of hospital stay (days)	ICU	VAD	MV	Therapeutics
1	RT-PCR	<50%	Yes	4	Yes	No	No	Azithromycin
2	RT-PCR	>50%	Yes	4	Yes	Yes	Yes	HCQ, Azithromycin, CE
3	RT-PCR	<50%	No	11	No	No	No	NP
4	Chest CT	<50%	No	5	No	No	No	Azithromycin
5	RT-PCR	<50%	No	21	No	No	No	NP

RT-PCR: polymerase chain reaction for SARS-CoV-2; ICU: Intensive Care Unit; VAD: vasoactive drug; MV: mechanical ventilation; HCQ: hydroxychloroquine; CE: corticosteroid; NP: not performed; CT: computed tomography.

Immunosuppressants were discontinued in one patient due to the severity of the case. Two patients died (40%), and the rest were discharged from hospital. The length of stay varied between 4 and 21 days.

## Discussion

This is the first description of a case series of a cohort of HT patients who were hospitalized by Covid-19 in Brazil.

These patients appear to present Covid19 similarly to non-transplanted patients, sharing the most common symptoms of fever, cough, and shortness of breath. In contrast to the study by Scott,^9^ gastrointestinal symptoms were observed in only one patient (20%).

All HT recipients that were affected by Covid-19 required hospitalization, with DM being present in 100% of them and SAH in 80%. In our sample, 40% of patients required intensive care and had D-Dimer ≥1000 mg/L and CRP ≥20. BNP was high and DHL did not show any significant increase when measured. The patient with elevated troponin presented hemodynamic instability, need for vasopressors and evolution to death, which corroborates the literature that associates myocardial injury with worse prognosis.^10^ The patients who went to the ICU were elderly, with a longer heart transplantation period, and died. These data suggest a transplant mortality rate above that of the general population infected by Covid-19.^11^

The rates of lymphopenia and thrombocytopenia were higher in this study when compared with previous reports in the non-transplanted and transplanted populations.^12^^,^^13^ This finding could be explained by a lower basal lymphocyte and platelet count due to the use of immunosuppressants or represent a likely additional interference from SARS-CoV-2 infection.

Our study has several limitations common to HT studies: the fact that it was performed in a single center and the size of our sample, which could be considered small. It was not possible to draw conclusions about specific treatments for Covid-19 or the management of immunosuppression in this scenario. This limits our understanding of the spectrum of symptoms and the severity of the disease among HT patients with Covid-19.

## Conclusion

In this case series of HT patients with Covid-19 treated at our institution, the theoretical possibility that immunosuppression could revoke the hyperinflammatory syndrome was not proven true. From an observational point of view, the large number of risk factors and the high mortality rate suggest that these receptors could be particularly vulnerable to Covid-19. Further larger, multicenter studies are needed to confirm our findings.
